# Social media impact on clear aligner decisions: A questionnaire study

**DOI:** 10.6026/973206300221164

**Published:** 2026-02-28

**Authors:** Neeraj Eknath Kolge, Priyanshi Chhajwani, Akshada Kapse, Abhishek Israni, Diksha Vinod Wali, Prachi Tushar Khatri, Zaineb Farooque, Shivani Tupdale

**Affiliations:** 1 Department of Orthodontics & Dentofacial Orthopaedics , Bharati Vidyapeeth Deemed (to be) University Dental College & Hospital, Navi Mumbai, Maharashtra, India; 2 Department of Dentistry, MGM Dental College & Hospital, Navi Mumbai, Maharashtra, India; 3 Department of Orthodontics & Dentofacial Orthopaedics, Pacific Dental College & Research Centre, Udaipur, Rajasthan, India; 4 Department of Pediatric and Preventive Dentistry, DY Patil School of Dentistry, Navi Mumbai, Maharashtra, India; 5 Government Dental College & Hospital, Mumbai, Maharashtra, India

**Keywords:** Clear aligners, orthodontics, patient perception, social media, treatment decision-making

## Abstract

The growing influence of social media on adult patients' decision-making regarding clear aligner treatment has raised concerns about its impact on treatment expectations and satisfaction. Despite the rising interest in clear aligners, a gap exists between awareness and actual treatment uptake. The research highlights the significant role social media plays in shaping awareness, while professional recommendations and factors like price and convenience remain the primary determinants of treatment selection. This study advances knowledge by highlighting social media's influence on patient perceptions of clear aligner treatment and the continued importance of professional guidance.

## Background:

In recent years, there has been a growing demand for orthodontic treatment among adult patients, with research suggesting a preference for more visually appealing options [1]. Clear aligners, first introduced in the late 1990s with Invisalign emerging in 1997, revolutionized orthodontic treatment by providing a discreet alternative to traditional metal braces [2]. Their advantages, including enhanced aesthetics, improved oral hygiene, greater comfort and dietary flexibility, have contributed to their widespread adoption. Since 1998, clear aligner therapy has been extensively utilized in clinical practice and is regarded as a more aesthetically pleasing and comfortable option compared to fixed orthodontic appliances [3]. Social media has emerged as an indispensable and omnipresent tool in the dissemination of health-related information, significantly influencing consumer behavior and decision-making across various medical and dental domains, including orthodontic treatments [4]. The digital revolution has transformed the way individuals seek process and act upon healthcare information, with social media platforms serving as powerful mediums for shaping patient choices. Among contemporary orthodontic interventions, clear aligners have gained unprecedented popularity as a preferred alternative to traditional fixed appliances, largely due to their discreet nature and perceived convenience [5]. Digital advancements have refined their effectiveness, but the rise of direct-to-consumer brands raises concerns about professional oversight.

Understanding their evolution helps assess social media's role in shaping patient perceptions and choices [6]. In the era of digital connectivity, social media platforms such as Instagram, Facebook, TikTok and YouTube have become vital sources of patient education, marketing and peer influence. The extensive availability of user-generated content, influencer-driven endorsements and targeted direct-to-consumer marketing strategies has exponentially expanded the reach of orthodontic brands, thereby influencing consumer preferences and treatment choices [7]. The persuasive nature of visually compelling content, testimonials and before-and-after transformations has contributed to a significant shift in how patients perceive orthodontic treatment options. While social media provides accessibility to a wealth of information, the reliability and accuracy of such content remain areas of concern [8]. Therefore, it is of interest to determine the influence of social media on patient decision-making in clear aligner therapy, assessing both its positive and negative impacts and highlighting the need for professional oversight to ensure informed and realistic treatment choices.

## Materials and Methods:

This was a cross-sectional descriptive study carried out in 2025. A multiple-choice questionnaire containing 20 questions was distributed via Google forms amongst Orthodontic clinics and online survey platforms targeting individuals who have undergone or are considering clear aligner treatment. The respondents were reached using email and WhatsApp messenger. A total of 140 responses were collected. The questionnaire focused on determining whether social media platforms have an influence on the patients in their decision making and expectation of clear aligner treatment. The data was then organized in the form of pie-charts and graphs and was tabulated to calculate the percentage score for each question. Participation was voluntary and informed consent was obtained from all respondents prior to data collection. Responses were anonymized to maintain confidentiality. Data were compiled and subjected to statistical analysis. Descriptive statistics were used to summarize the responses in terms of frequencies and percentages. Chi-square tests were employed to assess the statistical significance of response distributions, with the level of significance set at p < 0.05. Statistical analysis was performed using standard statistical software (Table 1).

## Results:

A total of 140 responses were obtained through the questionnaire survey. The number of responses varied slightly across questions due to incomplete entries, ranging from 133 to 139 responses. Only 5.8% of respondents reported currently undergoing clear aligner treatment, while 47.8% had considered but not proceeded with treatment. An equal proportion (47.8%) had never considered clear aligner therapy. This distribution was statistically significant (χ^2^ = 48.00, p < 0.001). Dentists or orthodontists were reported as the primary source of information by 51.1% of respondents, followed by television or online advertisements (41.7%). Friends or family accounted for 7.2% of responses (χ^2^ = 44.80, p < 0.001). Frequent exposure to clear aligner advertisements on social media was reported by 64.7% of respondents, while 33.1% encountered them occasionally. Only 4.3% had never seen such advertisements (χ^2^ = 75.94, p < 0.001). Influencer or celebrity promotions influenced the opinions of 45.7% of respondents, whereas 39.1% reported no influence. 18.1% had not encountered such content (χ^2^ = 17.27, p < 0.001). Social media increased interest in clear aligner therapy among 48.9% of respondents, increased trust among 15.8% and increased skepticism among 11.5%. 29.5% reported no effect (χ^2^ = 46.64, p < 0.001). A majority (88.5%) reported greater trust in aligner brands when endorsed by dentists on social media (χ^2^ = 81.08, p < 0.001). Price (41.7%), convenience (30.9%) and dentist recommendation (27.3%) were the most important factors influencing treatment choice. Celebrity endorsements were not selected by any respondent (χ^2^ = 52.35, p < 0.001). More than half (51.9%) researched clear aligners on social media before decision-making (χ^2^ = 22.35, p < 0.001). Before-and-after photographs (52.6%) and treatment videos (50.4%) were considered the most helpful forms of content. A large majority (77.4%) would trust a brand more if it showed real customer reviews and feedback on social media. This underscores the power of social proof and authenticity in building consumer confidence. Almost half (46.4%) find social media ads convincing, with a similar proportion (42%) being unsure and only a small percentage (15.9%) find them not convincing. This suggests that social media ads generally have a positive or at least neutral impact on perceived convincingness.

Visual content, particularly videos (43.1%) and before-and-after photos (38.7%), are by far the most helpful types of social media content. Written reviews (8.8%), blog posts (7.3%) and Q & A sessions (2.2%) are significantly less impactful, reinforcing the importance of visual and dynamic content. Only 12.4% reported purchasing or seriously considering treatment due to social media advertisements. Following social media exposure, 68.8% felt more comfortable discussing clear aligners with their dentist. Before-and-after results were considered realistic by 44.9%, exaggerated by 21.7% and uncertain by 35.5% of respondents. A total of 24.6% of respondents reported frequently seeing posts related to treatment cost, while 34.1% reported occasional exposure. However, 45.7% of respondents indicated that they had not seen any cost-related posts on social media. Approximately 29.9% of respondents reported that such experiences would definitely influence their decision, whereas 45.3% stated they would decide independently. The remaining 26.3% were unsure about the influence. A majority (60.1%) have not considered booking a consultation directly through social media links/ads. While 15.2% have and 24.6% are unsure but might, this indicates that social media is more of an information and awareness platform rather than a direct booking channel for many. The most commonly desired information included step-by-step treatment guides (35.5%) and real user reviews (26.1%), followed by cost breakdowns and information on risks or downsides (15.2% each), while 8.0% preferred success or transformation stories. About 45.7% of respondents stated they would recommend clear aligners based on social media information, whereas 21.0% would not. A considerable proportion (37.7%) remained unsure, indicating mixed confidence levels (Table 2).

## Discussion:

The present questionnaire-based study evaluated the influence of social media on patient decision-making, expectations and perceptions regarding clear aligner therapy in Navi Mumbai. The findings demonstrate that while awareness and exposure to clear aligners through digital platforms are high, actual adoption remains comparatively low, indicating a significant gap between interest and treatment uptake. These observations are consistent with previous studies that report an increasing demand for aesthetically acceptable aligners and also demonstrate higher knowledge and a favourable perception towards clear aligner treatment [9]. A key factor in decision making for clear aligner therapy is the cost associated, although this study does not directly consider the relation of cost with treatment uptake it sheds light on the factor of cost of treatment, that can be broken down further into lack of oral health insurance, high out of pockets costs and lack of cost transparency that can affect decision making, the findings of this study also provide consistent, reliable observation highly suggestive of cost effectiveness as a factor in determination of aligner treatment uptake [10]. A key finding of this study was the dominant role of dental professionals in building trust, even within a social media-driven information environment.

Dental practice social media has proven to be far more effective in positively influencing treatment uptake as professional led vetted information with scientific backed knowledge has potential to counteract the hazards of misinformation and act as source of truth [11]. This supports previous literature emphasizing the importance of clinician involvement in patient education and treatment acceptance. Study by Al-Silwadi *et al.* (2015) [12] have similarly shown that while audio-visual and social media content improves knowledge and engagement, patients still rely on orthodontists for validation and final decision-making. The strong influence of visual content, particularly before-and-after photographs and procedural videos, observed in this study is consistent with prior analyses of orthodontic content on platforms such as Instagram, TikTok and YouTube. However, a substantial proportion of respondents expressed scepticism regarding the realism of such content, echoing concerns raised by Baxmann *et al.* (2024) [13] about the variable quality, accuracy and readability of online aligner information. This scepticism may reflect an emerging patient awareness of the limitations of aligner therapy, which are well documented in clinical literature regarding biomechanical predictability, attachment dependence and treatment outcomes.

Social media appears to function more as an informational and preparatory tool, empowering patients to engage in more informed discussions with their orthodontists, however the relatively low rate of direct consultation booking through social media suggests the low effectiveness in converting interest to clinical action, on the contrary factors such as facilities and technology, word of mouth recommendations and dentist's reputation are stronger contributors as compared to social-media [14]. Potential limitations of the findings of this study, is generalizability owing to the statistic, 33.7% of the population of India has access to social media. Social-media usage differs from country to country and the work should therefore be repeated elsewhere for reproducibility. This was a self-reported online survey study and is therefore subject to respondent bias. The use of a cross sectional design does not allow us to draw causal conclusion. Overall, this study contributes to the existing literature by providing region-specific, patient-centric evidence that clarifies the nuanced role of social media in orthodontic decision-making. Unlike studies focusing primarily on content analysis or clinical outcomes, the present research highlights the interaction between digital influence and professional trust. It underscores that social media is a powerful adjunct - but not a replacement - for clinician-guided decision-making. By identifying gaps in cost communication, expectation management and content credibility, this study emphasizes the need for ethically driven, dentist-led digital engagement to bridge the gap between online influence and evidence-based orthodontic care.

## Conclusion:

The study underscores social media's significant role in shaping patient awareness and expectations regarding clear aligners, though its impact on actual treatment decisions remains limited. Visual content heavily influenced expectations, while professional recommendations and cost considerations guided decision-making. Social media facilitated patient-doctor communication and could benefit from greater transparency to better align patient expectations with clinical outcomes.

## Figures and Tables

**Table 1:** Structured 20-item questionnaire assessing the influence of social media on patient decision-making and perceptions regarding clear aligner therapy

S.No.	Question	Answer Options
1	Have you ever used or considered using clear aligners?	- Yes, I use them now
		- Yes, I considered them but did not get them
		- No, I have never considered them
2	Where do you usually learn about clear aligners?	- Dentist/Orthodontist
		- Friends/Family
		- TV/Online Ads
		- Other
3	Have you seen clear aligner ads or promotions on social media?	- Yes, frequently
		- Yes, occasionally
		- No, never
4	Have you watched influencers or celebrities talk about clear aligners?	- Yes and it influenced my opinion
		- Yes, but it didn't change my opinion
		- No, I haven't seen any
5	If social media influenced your decision about clear aligners, how did it affect you?	- Made me more interested
		- Made me trust them more
		- Made me skeptical
		- No effect
6	Would you trust a clear aligner brand more if it was promoted by a dentist on social media?	- Yes
		- No
7	What is most important to you when choosing clear aligners?	- Price
		- Convenience (e.g., at-home treatment)
		- Recommendation from a dentist
		- Celebrity or influencer endorsements
8	Before deciding to try clear aligners, did you do any research about them on social media?	- Yes, I researched them online and on social media
		- No, I mostly asked friends or family
		- No, I didn't do any research before deciding
9	What type of content on social media helped you learn about clear aligners?	- Photos and before/after results
		- Videos showing the process
		- Reviews from influencers/celebrities
		- Reviews from other users
		- Expert advice from dentists or orthodontists
10	Would you be more likely to trust a clear aligner brand if it showed real customer reviews on social media?	- Yes
		- Maybe
		- No
11	Do you feel that clear aligner advertisements on social media are convincing?	- Yes
		- No
		- Maybe
12	What type of content about clear aligners do you find most helpful on social media?	- Videos (e.g., tutorials, reviews)
		- Before and after photos
		- Written reviews or testimonials
		- Q & A sessions or live chats
		- Informational blog posts/articles
13	Have you ever purchased or considered purchasing clear aligners because of an ad on social media?	- Yes
		- No
		- I'm still considering
14	After seeing content about clear aligners on social media, do you feel more comfortable discussing them with your dentist or orthodontist?	- Yes
		- No
		- I haven't discussed it with my dentist
15	Do you believe the results shown in before/after photos about clear aligners on social media are realistic?	- Yes, they seem realistic to me
		- No, they seem exaggerated or altered
		- I'm unsure
16	Have you seen social media posts talking about the cost of clear aligners?	- Yes, frequently
16	Have you seen social media posts talking about the cost of clear aligners?	- Yes, frequently
16	Have you seen social media posts talking about the cost of clear aligners?	- Yes, frequently
		- Yes, occasionally
		- No, I haven't seen any
17	If you saw a friend or family member using clear aligners based on social media recommendations, would that influence your decision to try them?	- Yes, it would definitely influence me
		- Maybe, I'd consider it more
		- No, I'll still make my own decision
18	What additional information would you like to see on social media about clear aligners?	- Step-by-step treatment guides
		- Reviews from real users
		- Flexible payment plans
		- Detailed cost breakdowns
		- Possible risks or downsides
		- Success stories and real-life transformations
19	Have you ever considered booking a consultation for clear aligners through social media ads or offers?	- Yes, I have done it
		- No, I haven't considered that
20	Would you recommend clear aligners to a friend or family member based on what you've seen on social media?	- Yes
		- No
		- I'm unsure

**Table 2:** Influence of social media on patient decision-making, perception, and behavioral intentions regarding clear aligner therapy (n = 140)

Variable	Response Category	n (%)	χ²	p-value
Clear Aligner Status	Currently undergoing treatment	8 (5.8)	48	<0.001
	Considered but not undertaken	67 (47.8)		
	Never considered	67 (47.8)		
Primary Source of Information	Dentist/Orthodontist	72 (51.1)	44.8	<0.001
	TV/Online advertisements	58 (41.7)		
	Friends/Family	10 (7.2)		
Exposure to Social Media Advertisements	Frequent	91 (64.7)	75.94	<0.001
	Occasional	46 (33.1)		
	Never	6 (4.3)		
Influencer/Celebrity Impact	Influenced opinion	64 (45.7)	17.27	<0.001
	No influence	55 (39.1)		
	Not encountered	25 (18.1)		
Effect of Social Media on Perception	Increased interest	69 (48.9)	46.64	<0.001
	Increased trust	22 (15.8)		
	Increased skepticism	16 (11.5)		
	No effect	41 (29.5)		
Trust if Endorsed by Dentist Online	Yes	124 (88.5)	81.08	<0.001
	No	16 (11.5)		
Most Important Treatment Selection Factor	Price	58 (41.7)	52.35	<0.001
	Convenience	43 (30.9)		
	Dentist recommendation	38 (27.3)		
	Celebrity endorsement	0 (0)		
Pre-treatment Social Media Research	Yes	73 (51.9)	22.35	<0.001
	No	67 (48.1)		
Trust if Real Reviews Displayed	Yes	108 (77.4)	'	'
	No/Unsure	32 (22.6)		
Are Social Media Ads Convincing?	Yes	65 (46.4)	'	'
	No	22 (15.9)		
	Unsure	59 (42.0)		
Purchased/Considered Due to Ads	Yes	17 (12.4)	'	'
	No/Still considering	123 (87.6)		
Comfort Discussing with Dentist After Exposure	Yes	96 (68.8)	'	'
	No	44 (31.2)		
Perceived Realism of Before/After Results	Realistic	62 (44.9)	'	'
	Exaggerated	30 (21.7)		
	Unsure	49 (35.5)		
Exposure to Cost-related Posts	Frequent	34 (24.6)	'	'
	Occasional	47 (34.1)		
	Never	63 (45.7)		
Influence of Peer Experience via Social Media	Definitely influence	42 (29.9)	'	'
	Independent decision	63 (45.3)		
	Unsure	37 (26.3)		
Booked Consultation via Social Media	Yes	21 (15.2)	'	'
	No	84 (60.1)		
	Unsure	35 (24.6)		
Recommendation Based on Social Media Information	Yes	64 (45.7)	'	'
	No	29 (21.0)		
	Unsure	53 (37.7)		
